# Exploring the Endocannabinoid System’s Influence on Mammary Tissue and Breast Milk Inflammation in Maternal Chronic Obesity

**DOI:** 10.3390/biom16020201

**Published:** 2026-01-28

**Authors:** Sebastián Elgueta, Natalia Sánchez, Pascuala Valdivia, Susana Contreras-Duarte

**Affiliations:** 1 School of Nutrition and Dietetics, Faculty of Rehabilitation and Quality of Life Sciences, Universidad San Sebastián, Santiago 7510602, Chile; sebastian.elgueta@uss.cl; 2 Department of Anatomy, Faculty of Medicine, Pontificia Universidad Católica de Chile, Santiago 8331150, Chile; nlsanche@uc.cl; 3 Cancer Cell Biology Laboratory, Centro de Biología Celular y Biomedicina (CEBICEM), Faculty of Medicine and Science, Universidad San Sebastián, Santiago 8420524, Chile; pvaldiviag@correo.uss.cl

**Keywords:** obesity, breast milk, inflammation, cannabinoid system, mammary gland, adipocytes

## Abstract

Approximately 40% of women start pregnancy with overweight or obesity, and around 70% retain weight in the postpartum period (PP). Obesity is related to low-grade systemic inflammation, primarily driven by the secretome of white adipose tissue (WAT), which includes dysfunctional adipocytes and immune cells that infiltrate WAT, releasing pro-inflammatory signals into the circulation. In women with obesity, the mammary gland undergoes structural and endocrine changes, such as reduced prolactin secretion, fewer mammary gland branches, and a higher abundance of adipocytes in mammary fat pad, which have not been studied under this condition. Maternal obesity during lactation also alters the composition of breast milk, promoting pro-inflammatory characteristics. The endocannabinoid system (ECS) is hyperactive in obesity, contributing to metabolic inflammation. Its primary receptors, cannabinoids 1 and 2, are expressed in the mammary gland and implicated in inflammation and weight gain. Endocannabinoids (ECs), the main ECS ligands, are typically not released into the bloodstream; however, their increased levels in obesity suggest upregulation in peripheral tissues. ECs are also present in breast milk, where their higher concentrations in women with obesity may influence infant food intake.

## 1. Introduction

Obesity is characterized by abnormal or excessive fat accumulation, considered to be a body mass index (BMI) > 30 Kg/m^2^ [1]. It is also characterized by low-grade systemic inflammation [2,3] and is recognized as a risk factor for developing cardiovascular disease (CVD) [4].

Worryingly, one-third of women enter the pregnancy period with obesity and remain with increased weight in the postpartum period (PP) [5,6,7]. These data suggest that obesity continues to be a concern after childbirth and may impact maternal and infant long-term health. In lactating women, obesity is linked to an increased amount of adipose mass [8], and the inflammation associated with weight gain or obesity is related to the secretion of pro-inflammatory mediators from adipose tissue [2,9]. Owing to the fundamental role of inflammation in the pathophysiology of obesity and the physiology of pregnancy, the combination of maternal adaptations and obesity-related inflammation may exacerbate and result in an elevation in inflammatory mediators.

White adipose tissue (WAT) constitutes most of the adipose tissue in the body and contains a variety array of cell types, within adipocytes being the most predominant [10]. Non-adipocyte cells in WAT are found in the stromal vascular fraction, with macrophages accounting for about 10% of these cells [10]. Macrophages present in WAT correlate directly with adiposity and the size of adipocytes in both humans and mice [11]. In obesity, macrophages can increase in number up to 40% of the adipose tissue mass [12]. These cells represent the main producers of TNF-α, produced by WAT, which contribute approximately 50% of IL-6- output [11,13] and produce the macrophage inflammatory protein 1 [2]. In addition, the blood levels of TNF-α, IL-1β, and IL-6 increase in proportion with adiposity [13,14].

Early in the development of obesity in humans, B lymphocytes [15] and T cells infiltrate adipose tissue, other immune cells, such as neutrophils, infiltrate transiently, and other protective immune cells become less abundant or are dysfunctional [16]. Additionally, a major influx of macrophages arrives at the adipose tissue [12,16]. Macrophage infiltration into adipose tissue, along with triglycerides accumulation, leads to their activation and tissue damage, a process that is similar to the formation of an atherosclerotic lesion [17,18]. This progressive shift in macrophage sub-type to an activated phenotype/pro-inflammatory, called M1 polarization [19], is stimulated by inflammatory factors released by fatty tissue such as interferon γ (IFN-γ) and monocyte chemoattractant protein-1 (MCP-1) [10], which contribute to chronic adipose tissue inflammation through the pro-inflammatory release of IL-6, IL-1β, IL-12, and TNF-α [10,20,21]. The activated macrophages promote the recruitment of additional macrophages from the circulation with pronounced M1 gene expression, forming crown-like structures (CLS) surrounding dying adipocytes, representing inflammation foci in this tissue [22,23,24]. Actually, in obese mice and humans, over 90% of the macrophage population found in WAT are near these cell remnants from adipocytes and form multinucleate giant cells, which are the hallmarks of chronic subclinical inflammation [25]. Obesity-induced macrophage accumulation within tissue and also occurs in mammary adipose tissue (MAT) [23,24,26].

**Figure 1:**
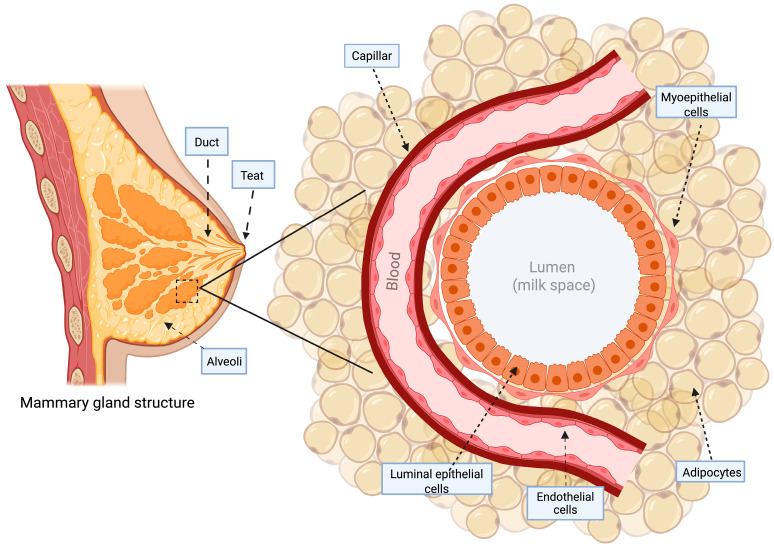
Mammary gland structure. The gland is structurally supported by the fat pad, with a network of capillaries. Alveoli are encased by myoepithelial cells, whose contraction facilitates milk ejection through ducts lined by luminal epithelial cells, responsible for milk secretion into the lumen. Created in BioRender. Gabriela Arenas. (2026). https://app.biorender.com/illustrations/660ed3c278d564b8620dd2eb (accessed on 20 November 2025).

The mammary gland is organized into a network of ducts and alveoli [27]. Architecturally, epithelial cells form the mammary gland ductal network of the gland [28]. Adipocytes dominate the stromal connective tissue, comprising the mammary fat pad that embeds and retains the ductal network, composed of a bilayered epithelial structure that facilitates the transport of nutrients, blood supply, and immune components via the vasculature [28], Figure 1.

Regarding the ductal network embedded in the mammary gland, the tissue is organized into two distinct cellular layers, with luminal epithelial cells constituting the inner layer that form the lactiferous duct, surrounding the central lumen that develops into alveoli or lobules, which are the secretory structures, responsible for milk production [29], and an external layer of basal epithelium composed of myoepithelial cells in direct contact with the basement membrane [28,30], comprising the basal layer of mature mammary ducts, which includes stem and progenitor cells responsible for generating the luminal and basal epithelial populations [29].

Throughout a woman’s life, the mammary gland tissue undergoes marked transformations [31], culminating in its full maturation during puberty and adulthood to carry out its essential role in lactation [30]. In this period, the mammary gland undergoes significant vascular expansion situated in the stroma compartment to facilitate milk production through the provision of micro- and macromolecules [28,32]. Luminal cells with a spindle-like morphology, oriented along the length of ducts and alveolar structures, mediate milk synthesis [33]. Myoepithelium cells, wrapping around epithelium alveolar structures, are responsible for ejecting milk [33]. Myoepithelial cells facilitate milk collection and ejection from the 15 to 20 lobular ducts, which merge into interlobular ducts, allowing each lobe to drain milk into the nipple [34]. Thus, neonatal suckling triggers the release of oxytocin from the neurohypophysis into the maternal bloodstream [33]. Oxytocin interacts with its receptor on alveolar basal cells in the vascularized mammary epithelium, triggering cell contraction [33], promoting milk movement towards the nipple [34]. Myoepithelial contraction creates a compressive force on the milk-secreting epithelium, pushing milk into the adjacent duct network [35]; additionally, this force stimulates a further release of lipid droplets from the luminal secretory cell population [36], by synchronizing emptying with refilling, it promotes sustained lactational output over time. Myoepithelial contractions along the longitudinal axis of the ducts complement the pressure from alveolar contraction to propel viscous milk through the ductal epithelium [33,35].

It is well established that obesity disrupts different processes related to mammary gland development and milk letdown [37,38]. In this regard, mammary luminal epithelial cells from women with OB are increased compared to women with a normal BMI [37,39]; this result was also found in mice exposed to a high-fat diet (HFD), broadly used as a model of obesity [40,41]. In addition, the enlarged mammary glands found in this model were attributed to an expansion of the adipose tissue [42,43], with a decreased quantity of ducts, narrower, with fewer branches [42,43], smaller alveoli [43], and with more collagen deposit with respect to lean mice [42], consequently leading to adipose tissue fibrosis [37]. Moreover, this murine model of obesity presents a reduction in the myoepithelial cell number [39,42], which likely contributes to impaired lactogenesis due to the retention of lipid accumulation in luminal cells [43]. This is a fact that is related to the low volume of breast milk observed in this model, even though the breast milk secretion improves on the second to third day of lactation [43]. With all these physiologic alterations, women with OB are linked to reduced rates of breastfeeding initiation [44,45] and shorter breastfeeding duration [46]. Women with OW/OB are more likely to discontinue breastfeeding before six months PP [47]. Similar to women with chronic obesity, who show delayed milk letdown or lactogenesis II [48,49], they tend to wean earlier than recommended [45].

Thus, obesity during the lactation period has many disadvantages for the dyad. Here, it is important to highlight the relevance of metabolic and weight maternal control before gestation and treatments that may help these women maintain lactation. This will ensure the mother’s benefits in decreasing weight during this period and benefits for the infant related to obtaining breast milk.

## 2. Review Methods

To address the main idea of this work, a comprehensive literature review was conducted using the Medline, ScienceDirect, and PubMed databases to search for articles published until July 2025. The search was performed using the following mesh terms and combinations of words: (“obesity” OR “overweight” OR “weight gain” OR “excessive weight gain”) AND (“pregnancy” OR “gestational” OR “postpartum” OR “puerperium”) AND (“inflammation” OR “cytokines” OR “immune cells” OR “macrophages” OR “lymphocytes” OR “crown like structures”) AND (“adipose tissue” OR “adipocyte” OR “dysfunctional adipocyte” OR “ white adipose tissue” OR “WAT”) AND (“mammary gland” OR “mammary gland structure” OR “mammary adipose tissue” OR “MAT”) AND (“milk let down” OR “ milk ejection” OR “prolactine” OR “oxitocyn”) AND (“breast milk” OR “breastmilk” OR “breastfeeding” OR “lactation” OR “lactogenesis” OR “maternal milk” OR “human milk” OR breast milk composition) AND (“endocannabinoid system” OR “endocannabinoids” OR “cannabinoids” OR “ anandamide” OR “AEA” OR “2-arachidonoylglycerol” OR “2-AG” OR “cannabinoid receptors” OR “CB1R” OR “ CB2R”). All mesh terms were combined as outlined in each described section. We selected full-length articles that performed studies in rats, mice, and humans (the main reviewed information is from humans). The electronic search was complemented by a manual review of the reference lists of selected publications and reviews on the issue to identify any other relevant publications.

### 2.1. Methodology Used to Quantify Endocannabinoids in the Breast Milk

Breast milk samples were prepared by adding 100 µL to microcentrifuge tubes and extracting lipids with 1 mL of methanol. Concentrations of AEA and 2-AG were measured using liquid chromatography–mass spectrometry (LC-MS), employing a surrogate analyte approach with deuterated standards AEA-d8 and 2-AG-d5. Calibration curves were generated by correlating peak areas of surrogate analytes to internal standards, analyzed on an AB Sciex QTRAP 5500 (Danaher Corporation, Toronto, ON, Canada) in positive ion mode. Finally, each sample and standard was injected in duplicate for analysis, with results averaging from triplicate measurements [50,51,52,53,54].

Inclusion and exclusion criteria: studies were eligible if they offered quantitative data on the associations between the previously mentioned mesh terms and if the articles were published in English.

### 2.2. Assessment of Study Quality and Risk of Bias

In this review, we conducted a qualitative assessment of the collected evidence in the included publications to estimate the methodological level and risk of bias. The included studies were classified according to their type as narrative review, systematic review, and observational or experimental studies, and their main methodological strengths and limitations were identified.

For human studies, prospective, cross-sectional, and cohort designs were distinguished, assessing the adjustment for confounding variables, sufficient sample size, and longitudinal analysis. For studies in animal models, adherence to the ARRIVE guidelines was reviewed, verifying the inclusion of criteria such as randomization, blinding, and ethics declaration. The articles included for this analysis were categorized as low, moderate, or high risk of bias, based on the criteria of methodological transparency, control of variables, and validity of conclusions.

## 3. Mammary Adipose Tissue Inflammation and Crown Like-Structure Development

Mediators produced in MAT have a paracrine role [55], and in the mammary stroma, the interaction of adipocytes forming the fat pad is essential in forming normal mouse mammary ducts [56]. Milk production relies on the local presence of functional adipocytes within the mammary gland, independent of their interaction with the epithelial compartment (which is primarily essential for the mammary gland formation). Additionally, adequate lactation requires a functional hormonal milieu, shaped by a healthy adipose depot that responds appropriately to its signals [57]. In fact, the selective removal of adipocytes decreased milk production [58], suggesting that adipocytes are essential for both milk maintenance and production.

In normal weight women, CLS are present [59], and it has been shown that environmental air pollution and race have an impact on the development of these structures [60]. In OW/OB women, a sub-inflammatory state of MAT promotes a characteristic dying adipocyte core surrounded by macrophages forming CLS, showing a significative odds ratio of 3.2- and 6.9 of forming CLS, respectively [55]. Interestingly, a modest enlargement of adipocytes is noted in some cases of CLS, suggesting that MAT inflammation can be initiated during early stages of hypertrophy [55]. Once breast adipocytes reach a threshold size, they undergo cell death, which initiates macrophage infiltration, a process observed as well in adipose tissue from alternative sites [22,23,24,25,26,57,61]. MAT inflammation is present across a range of adiposity, from moderate to excessive fat accumulation [55], and is commonly associated in individuals displaying visceral obesity [55]. The presence of CLS positive for IL-6 and for CD68 (a marker of macrophages) was linked with metabolic markers including glucose/(HbA1c) glycated hemoglobin, triglyceride/high-density lipoprotein-cholesterol ratio, and serum CRP, highlighting their pro-inflammatory status [55].

A healthy MAT is related to a healthy mammary adipocyte secretome, which is composed of growth factors that stimulate cell proliferation [62], angiogenic factors to achieve fat expansion, anti-inflammatory mediators like IL-10, transforming growth factor (TGF)-β, and pro-inflammatory cytokines such as IL-6, IL-8, and tumor necrosis factor (TNF)-α [63]. Hypertrophic and dysfunctional adipocytes in obesity secrete pro-inflammatory profile, including cytokines such as TNFα, IL-1β, and IL-6, chemokines (e.g., CCL2 and CCL5) recruiting M1 polarized inflammatory macrophages, and altered extracellular matrix components [64,65]. In addition, the obese mammary gland fat pad is enriched in myofibroblasts, contributing to the deposition of fibrillar and rigid extracellular matrix [64,66], producing high levels of collagen VI responsible for hypoxia [67] and, therefore, inflammation [64,68]. However, currently, it has not been evaluated how the mammary gland adipose tissue secretome can affect BM composition, with a special focus on pro-inflammatory metabolic diseases such as obesity.

## 4. Inflammatory Characteristics of Breast Milk Derived from Overweight and Obese Women

BM remains the gold standard for meeting the nutritional needs of newborns [69]. According to the WHO, the exclusive consumption of BM in the first half year of the newborn’s life is recommended to obtain its most favorable outcomes [70]. This biofluid is complex and contains a diverse array of bioactive components—both nutritional and regulatory—such as enzymes, hormones, and immune factors like chemokines, cytokines, immunoglobulins, and immune cells [71]. Its composition has adapted to provide essential nutrients and immune support to regulate the offspring’s growth, development, and metabolism [72,73]. Classically, infants who receive BM exhibit decreased mortality rates, better development, a lower rate of chronic diseases, a lower prevalence of gastrointestinal pathologies [74], and a diminished burden of overweight and obesity in the dyad 2–5 years PP [75].

During pregnancy, the lactation period is also a critical window through which BM mediates the transfer of biological signals from the mother to the infant [76], a fact that can determine the development and future health of the offspring and, thus, is a significant factor in both the risk and prevention of chronic diseases [77,78]. In addition, the composition of BM changes during the lactation period; it is biologically adapted to the infant according to its growth requirements, and its bioactive components vary from one woman to another [79]. The BM components are evolutionary tailored to support optimal infant growth; they can be affected by the maternal diet and health [76]. Evidence from human and animal studies suggests that maternal exposures can influence the BM composition [71]. In particular, the functional consequence of maternal pathologies such as obesity on BM composition is an area that needs further and deeper exploration.

Among the scarce information that has been published regarding this topic, most of the research has been conducted by mixing two groups of women, i.e., overweight (OW) and women with obesity (OB). Even more, regarding the last group, there is little distinction as to the type of obesity that these women have, i.e., pre-gestational obesity or obesity developed due to excessive weight gain. Thus, it is relevant to understand the proper contribution of each nutritional stage to the BM alteration.

An altered BM composition has been described in lactating mothers with OW and OB [8], characterized by higher levels of total fat content in mature milk, as supported by a meta-analysis [80], along with findings extending to six months PP [81]. In addition, BM from OW/OB women showed high levels of glucose [82,83], lactose [80], and insulin at 6 weeks [82,83] and 9 months PP [81].

In women with high BMI, elevated levels of lactoferrin- a key immune-modulating and anti-inflammatory protein-have been observed 15 days PP [84]. Additionally, an increased proportion of pro-inflammatory omega (n)-6 to omega (n)-3 polyunsaturated fatty acids has been reported in the colostrum from OW/OB women [85], as well as in mature milk from women with obesity [86,87]. These alterations are accompanied by reduced levels of omega-3 fatty acids [88] and increased saturated fatty acid content in the same population [89]. C Reactive protein (CRP) is a protein synthesized by hepatic cells and increases with inflammation in the body. Increased BM concentrations were found among women with obesity and EWG during 1 to 3 months PP [90,91], as well as in OW/OB women from 1 to 9 months PP [81].

Some classical inflammation-related cytokines such as BM IL-6 in these studies, did not change their concentration in women with obesity or EWG 1 to 3 months PP [91] or in women with OW/OB [92,93]. In addition, no changes in INF-γ [93] and TNF-α levels [92,93] have been reported in women with elevated BMI.

Regarding the anti-inflammatory factors of BM, this fluid exhibits reduced circulation of anti-inflammatory cytokines, such as TGF-β 1 month PP in high BMI women [93], with no changes in IL-10 [93].

However, despite the characterization described regarding the alterations found in BM in the mentioned population, there is scarce information regarding the specific contribution of each nutritional status (i.e., OW/OB) to the altered BM profile or whether these described alterations are a synergic effect produced when these different women are gathered in one group. It is important to establish this issue so that recommendations can be classified and made accordingly. Moreover, it is important to understand whether lactation on its own or combined with pregnancy contributes to the obesity described in the offspring.

## 5. Endocannabinoid System as a Source of Inflammation in Obesity

The endocannabinoid system (ECS) constitutes a complex cellular signaling system present in humans and other mammalian species [94]. This system plays crucial functions in the physiological regulation of the body, essential in maintaining homeostasis and optimizing its functioning [94]. It regulates key variables such as the body temperature, acid–base balance, and blood glucose concentration [95]. The ECS influences the regulation of sleep and wakefulness, affecting the quality and duration of sleep phases [96]. It is a key mediator in stress-related pathways, modulating the release of related hormones and providing anxiolytic effects [97]. The modulation of pain at both central and peripheral levels of the nervous system is mediated by cannabinoid receptors, with CB1R playing a predominant role [98,99,100]. They impact synaptic plasticity and memory, modulating memory consolidation and learning processes [101,102]. They also modulate the production and release of immune mediators and the migration of immune cells, crucial for managing inflammatory conditions [103]. In addition, the ECs are also involved in regulating appetite and energy metabolism, with the activation of CB_1_R in the hypothalamus influencing food intake and energy balance [104]. These functions reflect this system’s broad scope and integral role in regulating numerous aspects of physiological functioning, from the molecular level to the nervous system, including central and peripheral tissues, highlighting its importance in overall health and well-being.

The ECS consist of endocannabinoids (ECs), their receptors, and proteins involved in EC transport, synthesis, and degradation [105,106]. ECs are endogenous compounds synthesized by the body, and the most extensively studied are anandamide (AEA), also known as arachidonoyl ethanolamine, and 2-arachidonoylglycerol (2-AG) [106]. These are derived from arachidonic acid, an omega-6 polyunsaturated fatty acid with a long carbon chain [107]. Synthesis of AEA is performed by its biosynthetic intermediate N-arachidonoyl phosphatidylethanolamine (NAPE) through cleavage by NAPE-phospholipase D in a calcium-dependent fashion [108,109]. Additionally, there are two other mechanisms independent of calcium action by the combined action of a/b-hydrolase 4 and glycerophosphodiesterase 1, which are less studied [108,109]. On the other hand, 2-AG is a product of the membrane phospholipids hydrolysis through phospholipase C (PLC) and DAGLa or DAGLb [110,111]. There are other ECs that are less abundant, such as palmitoylethanolamide (PEA), which is a lipid mediator with anti-inflammatory functions [112], and oleoylethanolamide (OEA), which is a natural antagonist of AEA, suppressing appetite [113].

**Figure 2:**
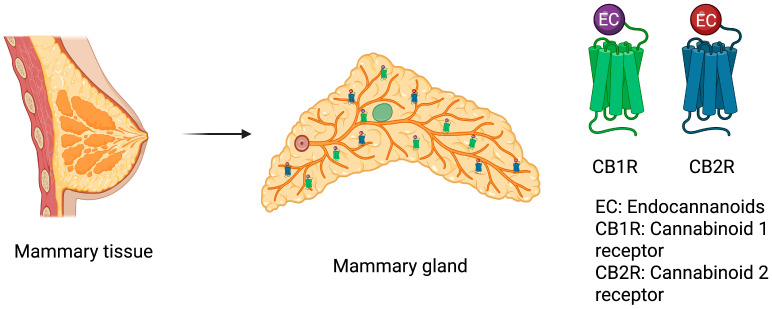
Cannabinoid receptors in the mammary gland tissue. Cannabinoid 1 and 2 receptors are distributed into the stroma and the fat pad in the mammary tissue. Created in BioRender. Susana Contreras. (2026). https://app.biorender.com/illustrations/696e64945194eccf68dc9aba (accessed on 20 November 2025).

Once the ECs are synthesized, they bind to two main types of ECs receptors associated to G proteins: CB_1_R and CB_2_R [106]. Although the brain is the main location of CB_1_R [106], this receptor is likewise distributed in non-central tissues [114], among which are the liver [115], as well as the gastrointestinal system [116,117], skeletal muscle [117], cardiovascular system [118], adipocytes [119], and the reproductive system [120], including adipose and epithelial components of the mammary tissue [121,122], Figure 2**.**

These ECs act as chemical messengers, released by adipose cells to activate CB1R [123], initiating intracellular events mainly through the GPCR pathway [106] and promoting energy conservation and food intake [124]. In WAT, ECs have effects that include decreasing leptin secretion [125], reducing lipolysis [126], increasing lipid storage capacity, increasing adipogenesis, and decreasing preadipocyte formation [127]. In this tissue, CB_1_R signaling has likewise been linked to inflammatory responses, the induction of insulin resistance, and relevant factors in the onset of metabolic conditions such as obesity [128]. In rat, when pharmacology blockade of this receptor is performed, it has been seen that it mitigates obesity-associated inflammation in extra-mammary tissues [129]. Meanwhile, in brown adipose tissue, CB_1_R decreases thermogenesis [130].

Additionally, CB_1_R signaling modulates energy balance, thus modulating appetite and metabolic energy use [128]. Central nervous system signaling modulates appetite and regulates body weight [131]. The ubiquitous location of CB_1_R highlights the broad influence of the ECS on diverse body organs and functions, evidencing its multifaceted role in regulating homeostasis [132].

In contrast, CB_2_R is primarily associated with immune system cells, and it is also present in tissues such as the spleen [133] and mammary glands (glandular cells and myoepithelial cells [121,122]). The activation of this receptor can significantly influence the function of cells in the immune system, providing an important mechanism in modulating the inflammatory cascade in various diseases [134]. This distinctive role of the CB_2_R underscores its importance in regulating the immune system and highlights its relevance as a therapeutic target for modulating inflammatory responses [135]. Under CB_2_R overactivation, Akt is activated, and inflammation is produced through NF-kB transcription [136].

Nevertheless, neither receptor has been studied during pregnancy or the lactation period, during which they may have different distributions and functions.

Specific degradation enzymes rapidly eliminate ECs free in the extracellular space [111]. The most studied enzymes that degrade ECs include fatty acid amide hydrolase (FAAH), which breaks down arachidonic acid and ethanolamine from AEA, and monoacylglycerol lipase (MAGL), which hydrolyzes 2-AG into arachidonic acid and glycerol, thereby finalizing EC degradation [111,137].

Another mechanism for regulating EC levels is adipokines [138]. In particular, leptin inhibits EC synthesis in white adipocytes, either through direct mechanism [138] or indirectly via its actions in the medio basal hypothalamus [138]. Insulin, akin to leptin, lowers EC concentration and enhances FAAH expression [139]. Nevertheless, obesity disrupts these negative loops regulating the ECS, which is characterized by resistance to the actions of both leptin and insulin [138].

Since ECs are not physiologically released from tissues into the bloodstream [140], elevated levels of them in the circulation likely suggest an upregulation of their synthesis in the peripheral organs due to obesity or degradation by their degradative enzymes [140]. During persistent disruptions, the endocannabinoid system undergoes regulatory imbalance, and its activity becomes persistently heightened [128,141], i.e., resulting in a loss of temporal and spatial precision in EC production and function [105], and cannabinoid receptors exhibit heightened activity (by upregulation) or are stimulated in cells that were not physiological targets of ECs [140]. This overactivation leads to increased visceral fat accumulation, weight increase, diminished adiponectin secretion from fat tissue, and the emergence of multiple obesity-related cardiometabolic risk factors [105].

Currently, it is undetermined whether mammary gland tissue with its endocannabinoid components, which likely process them, suffers similar alterations due to obesity.

## 6. Endocannabinoid Components in the Mammary Gland, Breast Milk, and Obesity

The ECS is present in the mammary gland, where the breast milk globule is assembled; understanding how these ECS components interact in the mammary gland can shed light on the adaptative mechanisms during lactation and underscore their implications for maternal health and infant development in the face of the obesity epidemic.

In this regard, CB_1_R and CB_2_R are found in human mammary glands [121,122,142]. In non-lactating mammary glands, CB_1_R is highly expressed in mammary gland adipocytes, presents low abundance in glandular cells, and is not expressed in myoepithelial cells in this tissue [121,122]; despite its distribution, its physiological role in this tissue is undetermined. In addition, in humans, CB_1_R interacts with dopamine receptor 2 (DR2), which, during lactation, participates in the programmed cell death of mammary epithelial cells and the regulation of milk protein production [143]. On the other hand, CB_2_R receptor has not been detected in non-lactating mammary gland adipocytes, but it is highly expressed in glandular cells and mildly expressed in myoepithelial cells [121,122], while both receptors have been found in epithelial cells [142]. CB_2_R has described 22 interactions with transcription factors, transporters, enzymes, and proteins such as adiponectin, that are present in breast tissue, as well in glandular and myoepithelial cells, with high and medium expression, respectively [121,122].

One of the main degraders of the AEA metabolite is FAAH, which is also found in glandular and mammary gland myoepithelial cells, with high and medium expression, respectively [121,122]. This enzyme drives lactogenic differentiation in vitro (through suppression of endocannabinoid tone and CB1R activity) and in vivo (being essential for the development of hormone-responsive luminal cells in the mammary gland) [144].

Some endocannabinoid system components have been shown to be detected in both mammary glands and in human BM [50,145,146,147,148]. The first description of this was measured in reproductive fluids, including mature breast milk, of ten women three months PP [147,148,149]. In mature breast milk, the concentration of anandamide was lower than the levels found of 2-AG [148,149], PEA, and OEA [145,146,147].

Besides the presence of ECs in breast milk, these lipids also change their concentration with the lactation stages [148]. In 2-week PP samples, the concentrations of 2-AG and AEA increased by 150% with respect to the 50% in samples older than 2 weeks [148], likely suggesting an impact on infant development [50] or a role in infant growth linked to weight gain. In fact, 2-AG and AEA concentrations increase in mature BM among women with elevated BMI [51,52]. Thus, it remains to be studied whether the alteration in the EC levels present in women who have obesity, which is presented in their breast milk during the PP, might be related to the infant’s weight gain described in the literature.

Endocannabinoid levels in human milk vary over the PP, with glycerol derivatives (e.g., 2-AG) showing time-dependent fluctuations. Ethanolamides, such as AEA, remain low [50,53,54].

Moreover, the endocannabinoid system seems to play a key role during the initial phases of the PP, as the administration of a CB1R antagonist (SR141716A) to mouse pups significantly decreases the milk suckling behavior on day 1 PP or daily from day 2 PP in a reversible manner [150]. However, the impact of ECS overactivation, seen in obesity, on lactation programming has been neglected.

## 7. Conclusions and Future Perspectives

Overweight, obesity, and gestational weight gain affect nursing women’s health and their breast milk quality. The mechanisms associated with these alterations remain to be elucidated. However, the inflammation produced by weight increment impairs adipocyte signaling in peripheral tissue, including the mammary gland adipose tissue. Emerging finding suggest that the endocannabinoid system may play a role in this process by modulating inflammatory responses within the mammary gland tissue potentially modifying the breast milk quality.

### 7.1. Future Perspectives

Obesity during lactation presents significant challenges for both mothers and infants, highlighting the essential need for maternal metabolic and weight management before gestation, along with interventions that support successful lactation. Effective strategies may facilitate maternal metabolic improvement during the breastfeeding period while ensuring infants receive the benefits of breast milk. Despite existing documentation on alterations in BM within this population, there is a lack of specific insights regarding the influence of various nutritional statuses, such as overweight and obesity, on these changes. It remains unclear whether these alterations are due to a synergistic effect when women of differing nutritional statuses are analyzed collectively. Addressing this uncertainty is vital for formulating tailored recommendations. Furthermore, understanding the role of lactation in the development of obesity in the offspring requires deeper exploration. Interestingly, the role of the ECS has been poorly evaluated during breastfeeding, while levels of these metabolites are largely described. Thus, research is needed to investigate whether the endocannabinoid levels in breast milk from women with chronic obesity while breastfeeding are associated with the weight gain of infants, as suggested in the literature. Moreover, the implications of ECS overactivation related to obesity on lactation programming have yet to be examined, indicating a crucial avenue for future research.

### 7.2. Limitations

The current review is mainly based on associative human data and mechanistic studies in animal models. As such, causal relationships, particularly between ECS dysregulation and infant metabolic outcomes, remain speculative. Further longitudinal and interventional research is required to clarify these mechanisms and their relevance to human lactation physiology.

## Data Availability

No new data were created or analyzed in this study.

## References

[1] Purnell J.Q. (2023). Definitions, Classification, and Epidemiology of Obesity. Endotext.

[2] Fantuzzi G. (2005). Adipose Tissue, Adipokines, and Inflammation. J. Allergy Clin. Immunol..

[3] Khanna D., Khanna S., Khanna P., Kahar P., Patel B.M. (2022). Obesity: A Chronic Low-Grade Inflammation and Its Markers. Cureus.

[4] Powell-Wiley T.M., Poirier P., Burke L.E., Després J.P., Gordon-Larsen P., Lavie C.J., Lear S.A., Ndumele C.E., Neeland I.J., Sanders P. (2021). Obesity and Cardiovascular Disease: A Scientific Statement from the American Heart Association. Circulation.

[5] Spencer L., Rollo M., Hauck Y., MacDonald-Wicks L., Wood L., Hutchesson M., Giglia R., Smith R., Collins C. (2015). The Effect of Weight Management Interventions That Include a Diet Component on Weight-Related Outcomes in Pregnant and Postpartum Women: A Systematic Review Protocol. JBI Database Syst. Rev. Implement. Rep..

[6] Lim S., Harrison C., Callander E., Walker R., Teede H., Moran L. (2022). Addressing Obesity in Preconception, Pregnancy, and Postpartum: A Review of the Literature. Curr. Obes. Rep..

[7] MINSAL (2019). Nutrición y Alimentación en la Gestante.

[8] Álvarez D., Muñoz Y., Ortiz M., Maliqueo M., Chouinard-Watkins R., Valenzuela R. (2020). Impact of Maternal Obesity on the Metabolism and Bioavailability of Polyunsaturated Fatty Acids during Pregnancy and Breastfeeding. Nutrients.

[9] Hrolfsdottir L., Schalkwijk C.G., Birgisdottir B.E., Gunnarsdottir I., Maslova E., Granström C., Strøm M., Olsen S.F., Halldorsson T.I. (2016). Maternal Diet, Gestational Weight Gain, and Inflammatory Markers during Pregnancy. Obesity.

[10] Liang W., Qi Y., Yi H., Mao C., Meng Q., Wang H., Zheng C. (2022). The Roles of Adipose Tissue Macrophages in Human Disease. Front. Immunol..

[11] Weisberg S.P., McCann D., Desai M., Rosenbaum M., Leibel R.L., Ferrante A.W. (2003). Obesity Is Associated with Macrophage Accumulation in Adipose Tissue. J. Clin. Investig..

[12] Longo M., Zatterale F., Naderi J., Parrillo L., Formisano P., Raciti G.A., Beguinot F., Miele C. (2019). Adipose Tissue Dysfunction as Determinant of Obesity-Associated Metabolic Complications. Int. J. Mol. Sci..

[13] Yao J., Wu D., Qiu Y. (2022). Adipose Tissue Macrophage in Obesity-Associated Metabolic Diseases. Front. Immunol..

[14] Cottam D.R., Mattar S.G., Barinas-Mitchell E., Eid G., Kuller L., Kelley D.E., Schauer P.R. (2004). The Chronic Inflammatory Hypothesis for the Morbidity Associated with Morbid Obesity: Implications and Effects of Weight Loss. Obes. Surg..

[15] Duffaut C., Galitzky J., Lafontan M., Bouloumié A. (2009). Unexpected Trafficking of Immune Cells within the Adipose Tissue during the Onset of Obesity. Biochem. Biophys. Res. Commun..

[16] Johnson A.R., Justin Milner J., Makowski L. (2012). The Inflammation Highway: Metabolism Accelerates Inflammatory Traffic in Obesity. Immunol. Rev..

[17] Xu H., Barnes G.T., Yang Q., Tan G., Yang D., Chou C.J., Sole J., Nichols A., Ross J.S., Tartaglia L.A. (2003). Chronic Inflammation in Fat Plays a Crucial Role in the Development of Obesity-Related Insulin Resistance. J. Clin. Investig..

[18] Cagnina A., Chabot O., Davin L., Lempereur M., Maréchal P., Oury C., Lancellotti P. (2022). Atherosclerosis, an inflammatory disease. Rev. Med. Liege.

[19] Ruggiero A.D., Key C.C.C., Kavanagh K. (2021). Adipose Tissue Macrophage Polarization in Healthy and Unhealthy Obesity. Front. Nutr..

[20] Thrum S., Sommer M., Raulien N., Gericke M., Massier L., Kovacs P., Krasselt M., Landgraf K., Körner A., Dietrich A. (2022). Macrophages in Obesity Are Characterised by Increased IL-1β Response to Calcium-Sensing Receptor Signals. Int. J. Obes..

[21] Thomas D., Apovian C. (2017). Macrophage Functions in Lean and Obese Adipose Tissue. Metabolism.

[22] Maliniak M.L., Cheriyan A.M., Cheriyan A.M., Sherman M.E., Liu Y., Liu Y., Gogineni K., Gogineni K., Liu J., He J. (2020). Detection of Crown-like Structures in Breast Adipose Tissue and Clinical Outcomes among African-American and White Women with Breast Cancer. Breast Cancer Res..

[23] Maliniak M.L., Miller-Kleinhenz J., Cronin-Fenton D.P., Lash T.L., Gogineni K., Janssen E.A.M., McCullough L.E. (2021). Crown-Like Structures in Breast Adipose Tissue: Early Evidence and Current Issues in Breast Cancer. Cancers.

[24] Chang M.C., Eslami Z., Ennis M., Goodwin P.J. (2021). Crown-like Structures in Breast Adipose Tissue of Breast Cancer Patients: Associations with CD68 Expression, Obesity, Metabolic Factors and Prognosis. npj Breast Cancer.

[25] Cinti S., Mitchell G., Barbatelli G., Murano I., Ceresi E., Faloia E., Wang S., Fortier M., Greenberg A.S., Obin M.S. (2005). Adipocyte Death Defines Macrophage Localization and Function in Adipose Tissue of Obese Mice and Humans. J. Lipid Res..

[26] Faria S.S., Corrêa L.H., Heyn G.S., de Sant’Ana L.P., Almeida R.D.N., Magalhães K.G. (2020). Obesity and Breast Cancer: The Role of Crown-Like Structures in Breast Adipose Tissue in Tumor Progression, Prognosis, and Therapy. J. Breast Cancer.

[27] McNally S., Stein T. (2017). Overview of Mammary Gland Development: A Comparison of Mouse and Human. Methods in Molecular Biology.

[28] Biswas S.K., Banerjee S., Baker G.W., Kuo C.Y., Chowdhury I. (2022). The Mammary Gland: Basic Structure and Molecular Signaling during Development. Int. J. Mol. Sci..

[29] Visvader J.E. (2009). Keeping Abreast of the Mammary Epithelial Hierarchy and Breast Tumorigenesis. Genes Dev..

[30] Cristea S., Polyak K. (2018). Dissecting the Mammary Gland One Cell at a Time. Nat. Commun..

[31] Froń A., Orczyk-Pawiłowicz M. (2023). Understanding the Immunological Quality of Breast Milk in Maternal Overweight and Obesity. Nutrients.

[32] Andres A.C., Djonov V. (2010). The Mammary Gland Vasculature Revisited. J. Mammary Gland Biol. Neoplasia.

[33] Gieniec K.A. (2022). Mammary Gland Architecture under the Lens: How Visualising Tissues in 3D Provides Deeper Insights into Structure and Function. Biochim. Biophys. Acta—Mol. Cell Res..

[34] Howard B.A., Gusterson B.A. (2000). Human Breast Development. J. Mammary Gland. Biol. Neoplasia.

[35] Stevenson A.J., Vanwalleghem G., Stewart T.A., Condon N.D., Lloyd-Lewis B., Marino N., Putney J.W., Scott E.K., Ewing A.D., Davis F.M. (2020). Multiscale Imaging of Basal Cell Dynamics in the Functionally Mature Mammary Gland. Proc. Natl. Acad. Sci. USA.

[36] Masedunskas A., Chena Y., Stussman R., Weigert R., Mather I.H. (2017). Kinetics of Milk Lipid Droplet Transport, Growth, and Secretion Revealed by Intravital Imaging: Lipid Droplet Release Is Intermittently Stimulated by Oxytocin. Mol. Biol. Cell.

[37] Hillers-Ziemer L.E., Arendt L.M. (2020). Weighing the Risk: Effects of Obesity on the Mammary Gland and Breast Cancer Risk. J. Mammary Gland Biol. Neoplasia.

[38] Luzardo-Ocampo I., Dena-Beltrán J.L., Ruiz-Herrera X., Ocampo-Ruiz A.L., Martínez de la Escalera G., Clapp C., Macotela Y. (2023). Obesity-Derived Alterations in the Lactating Mammary Gland: Focus on Prolactin. Mol. Cell Endocrinol..

[39] Chamberlin T., D’Amato J.V., Arendt L.M. (2017). Obesity Reversibly Depletes the Basal Cell Population and Enhances Mammary Epithelial Cell Estrogen Receptor Alpha Expression and Progenitor Activity. Breast Cancer Res..

[40] Li J., Wu H., Liu Y., Yang L. (2020). High Fat Diet Induced Obesity Model Using Four Strains of Mice: Kunming, C57BL/6, BALB/c and ICR. Exp. Anim..

[41] Martins T., Castro-Ribeiro C., Lemos S., Ferreira T., Nascimento-Gonçalves E., Rosa E., Oliveira P.A., Antunes L.M. (2022). Murine Models of Obesity. Obesities.

[42] Kamikawa A., Ichii O., Yamaji D., Imao T., Suzuki C., Okamatsu-Ogura Y., Terao A., Kon Y., Kimura K. (2009). Diet-Induced Obesity Disrupts Ductal Development in the Mammary Glands of Nonpregnant Mice. Dev. Dyn..

[43] Flint D.J., Travers M.T., Barber M.C., Binart N., Kelly P.A. (2005). Diet-Induced Obesity Impairs Mammary Development and Lactogenesis in Murine Mammary Gland. Am. J. Physiol. Endocrinol. Metab..

[44] Jevitt C. (2009). Pregnancy Complicated by Obesity: Midwifery Management. J. Midwifery Women’s Health.

[45] Chang Y.S., Glaria A.A., Davie P., Beake S., Bick D. (2020). Breastfeeding Experiences and Support for Women Who Are Overweight or Obese: A Mixed-methods Systematic Review. Matern. Child. Nutr..

[46] Buckley S., Uvnäs-Moberg K., Pajalic Z., Luegmair K., Ekström-Bergström A., Dencker A., Massarotti C., Kotlowska A., Callaway L., Morano S. (2023). Maternal and Newborn Plasma Oxytocin Levels in Response to Maternal Synthetic Oxytocin Administration during Labour, Birth and Postpartum—A Systematic Review with Implications for the Function of the Oxytocinergic System. BMC Pregnancy Childbirth.

[47] Rassie K., Dhungana R.R., Mousa A., Teede H., Joham A.E. (2024). Maternal Metabolic Conditions as Predictors of Breastfeeding Outcomes: Insights from an Australian Cohort Study. Acta Obstet. Gynecol. Scand..

[48] Nommsen-Rivers L.A., Chantry C.J., Peerson J.M., Cohen R.J., Dewey K.G. (2010). Delayed Onset of Lactogenesis among First-Time Mothers Is Related to Maternal Obesity and Factors Associated with Ineffective Breastfeeding. Am. J. Clin. Nutr..

[49] Rasmussen K.M., Hilson J.A., Kjolhede C.L. (2001). Obesity May Impair Lactogenesis II. J. Nutr..

[50] Gaitán A.V., Wood J.A.T., Zhang F., Makriyannis A., Lammi-Keefe C.J. (2018). Endocannabinoid Metabolome Characterization of Transitional and Mature Human Milk. Nutrients.

[51] Datta P., Melkus M.W., Rewers-Felkins K., Patel D., Bateman T., Baker T., Hale T.W. (2021). Human Milk Endocannabinoid Levels as a Function of Obesity and Diurnal Rhythm. Nutrients.

[52] Pontes T.F., Reis G., Santos G.R.C., Pereira H.M.G., Kac G., Ferreira A.L.L., Trevenzoli I.H. (2025). Maternal Obesity and Excessive Gestational Weight Gain Influence Endocannabinoid Levels in Human Milk Across Breastfeeding: Potential Implications for Offspring Development. Nutrients.

[53] Wood J.T., Durham H.A., Vadivel S.K., Makriyannis A., Lammi-Keefe C.J. (2013). Postpartum Changes in the Endocannabinoid Metabolome of Human Breast Milk. FASEB J..

[54] Csatári-Kovács R., Röszer T., Csősz É. (2025). Comparative Analysis of Omega-3, Omega-6, and Endocannabinoid Content of Human, Cattle, Goat, and Formula Milk. Foods.

[55] Vaysse C., Lomo J., Garred O., Fjeldheim F., Lofteroed T., Schlichting E., McTiernan A., Frydenberg H., Husoy A., Lundgren S. (2017). Inflammation of Mammary Adipose Tissue Occurs in Overweight and Obese Patients Exhibiting Early-Stage Breast Cancer. npj Breast Cancer.

[56] Zangani D., Darcy K.M., Shoemaker S., Ip M.M. (1999). Adipocyte-Epithelial Interactions Regulate the in Vitro Development of Normal Mammary Epithelial Cells. Exp. Cell Res..

[57] Brenot A., Hutson I., Harris C. (2020). Epithelial-Adipocyte Interactions Are Required for Mammary Gland Development, but Not for Milk Production or Fertility. Dev. Biol..

[58] Gregor M.F., Misch E.S., Yang L., Hummasti S., Inouye K.E., Lee A.H., Bierie B., Hotamisligil G.S. (2013). The Role of Adipocyte XBP1 in Metabolic Regulation during Lactation. Cell Rep..

[59] Berger N.A. (2017). Crown-like Structures in Breast Adipose Tissue from Normal Weight Women: Important Impact. Cancer Prev. Res..

[60] Harris A.R., Hughes J.D., Lawrence W.R., Lenz P., Franklin J., Bhawsar P.M.S., Dorsey T.H., Rossi E.L., Pichardo C.M., Pichardo M.S. (2025). Neighborhood Environment, DNA Methylation, and Presence of Crown-Like Structures of the Breast. JAMA Netw. Open.

[61] Murano I., Barbatelli G., Parisani V., Latini C., Muzzonigro G., Castellucci M., Cinti S. (2008). Dead Adipocytes, Detected as Crown-like Structures, Are Prevalent in Visceral Fat Depots of Genetically Obese Mice. J. Lipid Res..

[62] Hausman D.B., DiGirolamo M., Bartness T.J., Hausman G.J., Martin R.J. (2001). The Biology of White Adipocyte Proliferation. Obes. Rev..

[63] Trayhurn P., Wood I.S. (2004). Adipokines: Inflammation and the Pleiotropic Role of White Adipose Tissue. Br. J. Nutr..

[64] Colleluori G., Perugini J., Barbatelli G., Cinti S. (2021). Mammary Gland Adipocytes in Lactation Cycle, Obesity and Breast Cancer. Rev. Endocr. Metab. Disord..

[65] Zhang Z., Scherer P.E. (2018). Adipose Tissue: The Dysfunctional Adipocyte—A Cancer Cell’s Best Friend. Nat. Rev. Endocrinol..

[66] Seo B.R., Bhardwaj P., Choi S., Gonzalez J., Eguiluz R.C.A., Wang K., Mohanan S., Morris P.G., Du B., Zhou X.K. (2015). Obesity-Dependent Changes in Interstitial ECM Mechanics Promote Breast Tumorigenesis. Sci. Transl. Med..

[67] Pasarica M., Gowronska-Kozak B., Burk D., Remedios I., Hymel D., Gimble J., Ravussin E., Bray G.A., Smith S.R. (2009). Adipose Tissue Collagen VI in Obesity. J. Clin. Endocrinol. Metab..

[68] Khan I.T., Bule M., Ullah R., Nadeem M., Asif S., Niaz K. (2019). The Antioxidant Components of Milk and Their Role in Processing, Ripening, and Storage: Functional Food. Vet. World.

[69] Kalarikkal S.M., Pfleghaar J.L. (2024). Breastfeeding. StatPearls.

[70] World Health Organization Obesity and Overweight. https://www.who.int/news-room/fact-sheets/detail/obesity-and-overweight.

[71] Eisha S., Joarder I., Wijenayake S., Mcgowan P.O. (2022). Nutritive Bioactive Components in Maternal Milk and Offspring Development: A Scoping Review Non-Nutritive Bioactive Components in Maternal Milk and Offspring Development: A Scoping Review. J. Dev. Orig. Health Dis..

[72] Wijenayake S., Martz J., Lapp H.E., Storm J.A., Champagne F.A., Kentner A.C. (2023). The Contributions of Parental Lactation on Offspring Development: It’s Not Udder Nonsense!. Horm. Behav..

[73] Dieterich C.M., Felice J.P., O’Sullivan E., Rasmussen K.M. (2013). Breastfeeding and Health Outcomes for the Mother-Infant Dyad. Pediatr. Clin. N. Am..

[74] Lyons K.E., Ryan C.A., Dempsey E.M., Ross R.P., Stanton C. (2020). Breast Milk, a Source of Beneficial Microbes and Associated Benefits for Infant Health. Nutrients.

[75] Mantzorou M., Papandreou D., Pavlidou E., Papadopoulou S.K., Tolia M., Mentzelou M., Poutsidi A., Antasouras G., Vasios G.K., Giaginis C. (2023). Maternal Gestational Diabetes Is Associated with High Risk of Childhood Overweight and Obesity: A Cross-Sectional Study in Pre-School Children Aged 2–5 Years. Medicina.

[76] Bode L., Raman A., Murch S., Rollins N., Gordon J. (2020). Understanding the Mother-Breastmilk-Infant “Triad”. Science.

[77] Edwards P.D., Lavergne S.G., Mccaw L.K., Wijenayake S., Boonstra R., Mcgowan P.O., Holmes M.M. (2021). Maternal Effects in Mammals: Broadening Our Understanding of Offspring Programming Offspring Phenotype Androgens Photoperiod Melatonin Microbiome Immunity Maternal Milk Exosomes MicroRNA. Front. Neuroendocrinol..

[78] Lucas A. (1998). Programming by Early Nutrition: An Experimental Approach. J. Nutr..

[79] Lokossou G.A.G., Kouakanou L., Schumacher A., Zenclussen A.C. (2022). Human Breast Milk: From Food to Active Immune Response with Disease Protection in Infants and Mothers. Front. Immunol..

[80] Leghi G.E., Netting M.J., Middleton P.F., Wlodek M.E., Geddes D.T., Muhlhausler B.S. (2020). The Impact of Maternal Obesity on Human Milk Macronutrient Composition: A Systematic Review and Meta-Analysis. Nutrients.

[81] Sims C.R., Lipsmeyer M.E., Turner D.E., Andres A. (2020). Human Milk Composition Differs by Maternal BMI in the First 9 Months Postpartum. Am. J. Clin. Nutr..

[82] Ahuja S., Boylan M., Hart S.L., Román-Shriver C., Spallholz J.E., Pence B.C., Sawyer B.G. (2011). Glucose and Insulin Levels Are Increased in Obese and Overweight Mothers’ Breast-Milk. Food Nutr. Sci..

[83] Cheema A.S., Stinson L.F., Rea A., Lai C.T., Payne M.S., Murray K., Geddes D.T., Gridneva Z. (2021). Human Milk Lactose, Insulin, and Glucose Relative to Infant Body Composition during Exclusive Breastfeeding. Nutrients.

[84] Houghton M.R., Gracey M., Burke V., Bottrell C., Spargo R.M. (1985). Breast Milk Lactoferrin Levels in Relation to Maternal Nutritional Status. J. Pediatr. Gastroenterol. Nutr..

[85] Hua M.C., Su H.M., Yao T.C., Liao S.L., Tsai M.H., Su K.W., Chen L.C., Lai S.H., Chiu C.Y., Yeh K.W. (2024). The Association between Human Milk Fatty Acid Composition in Mothers with an Elevated Body Mass Index and Infant Growth Changes. Clin. Nutr..

[86] Panagos P.G., Vishwanathan R., Penfield-Cyr A., Matthan N.R., Shivappa N., Wirth M.D., Hebert J.R., Sen S. (2016). Breastmilk from Obese Mothers Has Pro-Inflammatory Properties and Decreased Neuroprotective Factors. J. Perinatol..

[87] De La Garza Puentes A., Alemany A.M., Chisaguano A.M., Goyanes R.M., Castellote A.I., Torres-Espínola F.J., García-Valdés L., Escudero-Marín M., Segura M.T., Campoy C. (2019). The Effect of Maternal Obesity on Breast Milk Fatty Acids and Its Association with Infant Growth and Cognition—The PREOBE Follow-Up. Nutrients.

[88] Tekin-Guler T., Koc N., Kara-Uzun A., Fisunoglu M. (2023). The Association of Pre-Pregnancy Obesity and Breast Milk Fatty Acids Composition and the Relationship of Postpartum Maternal Diet, Breast Milk Fatty Acids and Infant Growth. Breastfeed. Med..

[89] Simon Sarkadi L., Zhang M., Muránszky G., Vass R.A., Matsyura O., Benes E., Vari S.G. (2022). Fatty Acid Composition of Milk from Mothers with Normal Weight, Obesity, or Gestational Diabetes. Life.

[90] Erliana U.D., Fly A.D. (2019). The Function and Alteration of Immunological Properties in Human Milk of Obese Mothers. Nutrients.

[91] Whitaker K.M., Marino R.C., Haapala J.L., Foster L., Smith K.D., Teague A.M., Jacobs D.R., Fontaine P.L., McGovern P.M., Schoenfuss T.C. (2017). Associations of Maternal Weight Status Before, During, and After Pregnancy with Inflammatory Markers in Breast Milk. Obesity.

[92] Young B.E., Patinkin Z.W., Pyle L., de la Houssaye B., Davidson B.S., Geraghty S., Morrow A.L., Krebs N. (2017). Markers of Oxidative Stress in Human Milk Do Not Differ by Maternal BMI But Are Related to Infant Growth Trajectories. Matern. Child Health J..

[93] Collado M.C., Laitinen K., Salminen S., Isolauri E. (2012). Maternal Weight and Excessive Weight Gain during Pregnancy Modify the Immunomodulatory Potential of Breast Milk. Pediatr. Res..

[94] Silver R.J. (2019). The Endocannabinoid System of Animals. Animals.

[95] Bazwinsky-Wutschke I., Zipprich A., Dehghani F. (2019). Endocannabinoid System in Hepatic Glucose Metabolism, Fatty Liver Disease, and Cirrhosis. Int. J. Mol. Sci..

[96] Murillo-Rodríguez E., Budde H., Veras A.B., Rocha N.B., Telles-Correia D., Monteiro D., Cid L., Yamamoto T., Machado S., Torterolo P. (2020). The Endocannabinoid System May Modulate Sleep Disorders in Aging. Curr. Neuropharmacol..

[97] Garani R., Watts J.J., Mizrahi R. (2021). Endocannabinoid System in Psychotic and Mood Disorders, a Review of Human Studies. Prog. Neuro-Psychopharmacol. Biol. Psychiatry.

[98] Bourke S.L., Schlag A.K., O’Sullivan S.E., Nutt D.J., Finn D.P. (2022). Cannabinoids and the Endocannabinoid System in Fibromyalgia: A Review of Preclinical and Clinical Research. Pharmacol. Ther..

[99] Finn D.P., Haroutounian S., Hohmann A.G., Krane E., Soliman N., Rice A.S.C. (2021). Cannabinoids, the Endocannabinoid System, and Pain: A Review of Preclinical Studies. Pain.

[100] Barrie N., Manolios N. (2017). The Endocannabinoid System in Pain and Inflammation: Its Relevance to Rheumatic Disease. Eur. J. Rheumatol..

[101] Kruk-Slomka M., Dzik A., Budzynska B., Biala G. (2017). Endocannabinoid System: The Direct and Indirect Involvement in the Memory and Learning Processes—A Short Review. Mol. Neurobiol..

[102] Lunardi P., de Souza L.W., dos Santos B., Popik B., de Oliveira Alvares L. (2020). Effect of the Endocannabinoid System in Memory Updating and Forgetting. Neuroscience.

[103] Almogi-Hazan O., Or R. (2020). Cannabis, the Endocannabinoid System and Immunity-the Journey from the Bedside to the Bench and Back. Int. J. Mol. Sci..

[104] Pagano C., Rossato M., Vettor R. (2008). Endocannabinoids, Adipose Tissue and Lipid Metabolism. J. Neuroendocrinol..

[105] Di Marzo V. (2008). The Endocannabinoid System in Obesity and Type 2 Diabetes. Diabetologia.

[106] Lu H.C., Mackie K. (2021). Review of the Endocannabinoid System. Biol. Psychiatry Cogn. Neurosci. Neuroimaging.

[107] Alvheim A.R., Malde M.K., Osei-Hyiaman D., Hong Lin Y., Pawlosky R.J., Madsen L., Kristiansen K., Frøyland L., Hibbeln J.R. (2012). Dietary Linoleic Acid Elevates Endogenous 2-AG and Anandamide and Induces Obesity. Obesity.

[108] Liu J., Wang L., Harvey-White J., Osei-Hyiaman D., Razdan R., Gong Q., Chan A.C., Zhou Z., Huang B.X., Kim H.Y. (2006). A Biosynthetic Pathway for Anandamide. Proc. Natl. Acad. Sci. USA.

[109] Bryk M., Chwastek J., Kostrzewa M., Mlost J., Pędracka A., Starowicz K. (2020). Alterations in Anandamide Synthesis and Degradation during Osteoarthritis Progression in an Animal Model. Int. J. Mol. Sci..

[110] Silvestri C., Di Marzo V. (2013). The Endocannabinoid System in Energy Homeostasis and the Etiopathology of Metabolic Disorders. Cell Metab..

[111] Maccarrone M., Di Marzo V., Gertsch J., Grether U., Howlett A.C., Hua T., Makriyannis A., Piomelli D., Ueda N., van der Stelt M. (2023). Goods and Bads of the Endocannabinoid System as a Therapeutic Target: Lessons Learned after 30 Years. Pharmacol. Rev..

[112] Clayton P., Subah S., Venkatesh R., Hill M., Bogoda N. (2023). Palmitoylethanolamide: A Potential Alternative to Cannabidiol. J. Diet. Suppl..

[113] Romano A., Coccurello R., Giacovazzo G., Bedse G., Moles A., Gaetani S. (2014). Oleoylethanolamide: A Novel Potential Pharmacological Alternative to Cannabinoid Antagonists for the Control of Appetite. BioMed Res. Int..

[114] Zou S., Kumar U. (2018). Cannabinoid Receptors and the Endocannabinoid System: Signaling and Function in the Central Nervous System. Int. J. Mol. Sci..

[115] Mallat A., Teixeira-Clerc F., Deveaux V., Manin S., Lotersztajn S. (2011). The Endocannabinoid System as a Key Mediator during Liver Diseases: New Insights and Therapeutic Openings. Br. J. Pharmacol..

[116] Farooqi T., Bhuyan D.J., Low M., Sinclair J., Leonardi M., Armour M. (2023). Cannabis and Endometriosis: The Roles of the Gut Microbiota and the Endocannabinoid System. J. Clin. Med..

[117] Schönke M., Martinez-Tellez B., Rensen P.C. (2020). Role of the Endocannabinoid System in the Regulation of the Skeletal Muscle Response to Exercise. Curr. Opin. Pharmacol..

[118] Rabino M., Mallia S., Castiglioni E., Rovina D., Pompilio G., Gowran A. (2021). The Endocannabinoid System and Cannabidiol: Past, Present, and Prospective for Cardiovascular Diseases. Pharmaceuticals.

[119] Myers M.N., Abou-Rjeileh U., Chirivi M., Parales-Girón J., Lock A.L., Tam J., Zachut M., Contreras G.A. (2023). Cannabinoid-1 Receptor Activation Modulates Lipid Mobilization and Adipogenesis in the Adipose Tissue of Dairy Cows. J. Dairy. Sci..

[120] Walker O.L.S., Holloway A.C., Raha S. (2019). The Role of the Endocannabinoid System in Female Reproductive Tissues. J. Ovarian Res..

[121] The Human Protein Atlas CNR2 Protein Expression Summary. https://www.proteinatlas.org/ENSG00000188822-CNR2.

[122] The Human Protein Atlas CNR1 Protein Expression Summary. https://www.proteinatlas.org/ENSG00000118432-CNR1.

[123] Jung K.M., Lin L., Piomelli D. (2022). The Endocannabinoid System in the Adipose Organ. Rev. Endocr. Metab. Disord..

[124] Rakotoarivelo V., Sihag J., Flamand N. (2021). Role of the Endocannabinoid System in the Adipose Tissue with Focus on Energy Metabolism. Cells.

[125] Tam J., Szanda G., Drori A., Liu Z., Cinar R., Kashiwaya Y., Reitman M.L., Kunos G. (2017). Peripheral Cannabinoid-1 Receptor Blockade Restores Hypothalamic Leptin Signaling. Mol. Metab..

[126] Sidibeh C.O., Pereira M.J., Lau Börjesson J., Kamble P.G., Skrtic S., Katsogiannos P., Sundbom M., Svensson M.K., Eriksson J.W. (2017). Role of Cannabinoid Receptor 1 in Human Adipose Tissue for Lipolysis Regulation and Insulin Resistance. Endocrine.

[127] Ruhl T., Karthaus N., Kim B.S., Beier J.P. (2020). The Endocannabinoid Receptors CB1 and CB2 Affect the Regenerative Potential of Adipose Tissue MSCs. Exp. Cell Res..

[128] Schulz P., Hryhorowicz S., Rychter A.M., Zawada A., Słomski R., Dobrowolska A., Krela-Kaźmierczak I. (2021). What Role Does the Endocannabinoid System Play in the Pathogenesis of Obesity?. Nutrients.

[129] Eid B.G., Neamatallah T., Binmahfouz L.S., Bagher A.M., Alamoudi A.J., Aldawsari H.M., Hanafy A., Hasan A., El-Bassossy H.M., Abdel-Naim A.B. (2023). Effects of the CB1 Receptor Antagonists AM6545 and AM4113 on Metabolic Syndrome-Induced Prostatic Hyperplasia in Rats. Biomol. Biomed..

[130] Krott L.M., Piscitelli F., Heine M., Borrino S., Scheja L., Silvestri C., Heeren J., Di Marzo V. (2016). Endocannabinoid Regulation in White and Brown Adipose Tissue Following Thermogenic Activation. J. Lipid Res..

[131] Jager G., Witkamp R.F. (2014). The Endocannabinoid System and Appetite: Relevance for Food Reward. Nutr. Res. Rev..

[132] Joshi N., Onaivi E.S. (2019). Endocannabinoid System Components: Overview and Tissue Distribution. Recent Advances in Cannabinoid Physiology and Pathology. Advances in Experimental Medicine and Biology.

[133] Jordan C.J., Xi Z.X. (2019). Progress in Brain Cannabinoid CB2 Receptor Research: From Genes to Behavior. Neurosci. Biobehav. Rev..

[134] Tortora C., Di Paola A., Argenziano M., Creoli M., Marrapodi M.M., Cenni S., Tolone C., Rossi F., Strisciuglio C. (2022). Effects of CB2 Receptor Modulation on Macrophage Polarization in Pediatric Celiac Disease. Biomedicines.

[135] Whiting Z.M., Yin J., de la Harpe S.M., Vernall A.J., Grimsey N.L. (2022). Developing the Cannabinoid Receptor 2 (CB2) Pharmacopoeia: Past, Present, and Future. Trends Pharmacol. Sci..

[136] Pellati F., Borgonetti V., Brighenti V., Biagi M., Benvenuti S., Corsi L. (2018). *Cannabis Sativa*, L. and Nonpsychoactive Cannabinoids: Their Chemistry and Role against Oxidative Stress, Inflammation, and Cancer. BioMed Res. Int..

[137] Maccarrone M. (2020). Missing Pieces to the Endocannabinoid Puzzle. Trends Mol. Med..

[138] Matias I., Belluomo I., Cota D. (2016). The Fat Side of the Endocannabinoid System: Role of Endocannabinoids in the Adipocyte. Cannabis Cannabinoid Res..

[139] Miralpeix C., Reguera A.C., Fosch A., Zagmutt S., Casals N., Cota D., Rodríguez-Rodríguez R. (2021). Hypothalamic Endocannabinoids in Obesity: An Old Story with New Challenges. Cell Mol. Life Sci..

[140] Di Marzo V. (2008). Targeting the Endocannabinoid System: To Enhance or Reduce?. Nat. Rev. Drug Discov..

[141] Fajardo L., Sanchez P., Salles J., Rigaudière J.P., Patrac V., Caspar-Bauguil S., Bergoglgio C., Moro C., Walrand S., Le Bacquer O. (2023). Inhibition of the Endocannabinoid System Reverses Obese Phenotype in Aged Mice and Partly Restores Skeletal Muscle Function. Am. J. Physiol. Endocrinol. Metab..

[142] Qamri Z., Preet A., Nasser M.W., Bass C.E., Leone G., Barsky S.H., Ganju R.K. (2009). Synthetic Cannabinoid Receptor Agonists Inhibit Tumor Growth and Metastasis of Breast Cancer. Mol. Cancer Ther..

[143] Han L., Lu S.N., Nishimura T., Kobayashi K. (2024). Regulatory Roles of Dopamine D2 Receptor in Milk Protein Production and Apoptosis in Mammary Epithelial Cells. Exp. Cell Res..

[144] Tundidor I., Seijo-Vila M., Blasco-Benito S., Rubert-Hernández M., Moreno-Bueno G., Bindila L., de la Rosa R.F., Guzmán M., Sánchez C., Pérez-Gómez E. (2023). Fatty Acid Amide Hydrolase Drives Adult Mammary Gland Development by Promoting Luminal Cell Differentiation. bioRxiv.

[145] Battista N., Sergi M., Montesano C., Napoletano S., Compagnone D., Maccarrone M. (2014). Analytical Approaches for the Determination of Phytocannabinoids and Endocannabinoids in Human Matrices. Drug Test Anal..

[146] Lam P.M.W., Marczylo T.H., Konje J.C. (2010). Simultaneous Measurement of Three N-Acylethanolamides in Human Bio-Matrices Using Ultra Performance Liquid Chromatography-Tandem Mass Spectrometry. Anal. Bioanal. Chem..

[147] Schuel H., Burkman L.J., Lippes J., Crickard K., Forester E., Piomelli D., Giuffrida A. (2002). N-Acylethanolamines in Human Reproductive Fluids. Chem. Phys. Lipids.

[148] Wu J., Gouveia-Figueira S., Domellöf M., Zivkovic A.M., Nording M.L. (2016). Oxylipins, Endocannabinoids, and Related Compounds in Human Milk: Levels and Effects of Storage Conditions. Prostaglandins Other Lipid Mediat..

[149] Di Marzo V., Sepe N., De Petrocellis L., Berger A., Crozier G., Fride E., Mechoulam R. (1998). Trick or Treat from Food Endocannabinoids?. Nature.

[150] Fride E., Ginzburg Y., Breuer A., Bisogno T., Di Marzo V., Mechoulam R. (2001). Critical Role of the Endogenous Cannabinoid System in Mouse Pup Suckling and Growth. Eur. J. Pharmacol..

